# Review of Nematode Interactions with Hemp (*Cannabis Sativa*)

**DOI:** 10.21307/jofnem-2022-002

**Published:** 2022-02-18

**Authors:** Ernest C. Bernard, Angel G. Chaffin, Kimberly D. Gwinn

**Affiliations:** 1 Entomology and Plant Pathology, The University of Tennessee, 370 Plant Biotechnology, Building, 2505 E J Chapman Drive, Knoxville, TN 37996-4560.; 2 Pope's Plant Farm, Maryville, TN.

**Keywords:** *Cannabis sativa*, Cyst nematodes, *Ditylenchus*, Hemp, *Heterodera humuli*, Host-parasite relationships, Management, *Meloidogyne*, Plant extracts, *Pratylenchus*, Review, Root chemistry, Root-knot nematodes, Stem nematodes

## Abstract

The many decades during which the cultivation of *Cannabis sativa* (hemp) was strongly restricted by law resulted in little research on potential pathogenic nematodes of this increasingly important crop. The primary literature was searched for hemp-nematode papers, resulting in citations from 1890 through 2021. Reports were grouped into two categories: (i) nematodes as phytoparasites of hemp, and (ii) hemp and hemp products and extracts for managing nematode pests. Those genera with the most citations as phytoparasites were *Meloidogyne* (root-knot nematodes, 20 papers), *Pratylenchus* (lesion nematodes, 7) and *Ditylenchus* (stem nematodes, 7). Several *Meloidogyne* spp. were shown to reproduce on hemp and some field damage has been reported. Experiments with *Heterodera humuli* (hop cyst nematode) were contradictory. Twenty-three papers have been published on the effects of hemp and hemp products on plant-parasitic, animal-parasitic and microbivorous species. The effects of hemp tissue soil incorporation were studied in five papers; laboratory or glasshouse experiments with aqueous or ethanol extracts of hemp leaves accounted for most of the remainder. Many of these treatments had promising results but no evidence was found of large-scale implementation. The primary literature was also searched for chemistry of *C. sativa* roots. The most abundant chemicals were classified as phytosterols and triterpenoids. Cannabinoid concentration was frequently reported due to the interest in medicinal *C. sativa*. Literature on the impact of root-associated chemicals on plant parasitic nematodes was also searched; in cases where there were no reports, impacts on free-living or animal parasitic nematodes were discussed.

*Cannabis* spp. (hemp) and their desirable products need little introduction. Plants have been dispersed by humans for millenia and have many uses (Clarke and Merlin, 2013). The presence and use of psychoactive chemicals in the flower buds of some selections led to widespread regulation in much of the world in the early twentieth century. Strict regulation began before the full recognition of the pathogenic roles of plant-parasitic nematodes on crop yield and quality. Consequently, the current body of knowledge on hemp-nematode host-parasite relationships is quite small (McPartland, 1996; McPartland et al., 2000; Wang, 2021). Publications on nematodes of hemp prior to the 1990s, and some afterwards, usually are reports of occurrence or association and provide little or no useful data on pathogenicity. The burgeoning interest in medicinal *Cannabis*, as well as increasing production of industrial and seed hemp (Żuk-Gołaszewska and Gołaszewski, 2020), requires a modern approach to the potential problems of pathogens and pests on crop health. Questions on the role of psychoactive, medicinal or other constituents in pest enhancement or suppression also must still be addressed in experimental studies.

The genus *Cannabis* (first domesticated in early Neolithic times in East Asia) has a complex history profoundly influenced by many centuries of human cultivation (Ren et al., 2021). It has been vigorously debated whether the cultivated forms are comprised of two species, *C. indica* and *C. sativa* (Clarke and Merlin, 2013, 2015, 2016) or one species (*C. sativa* with several subspecies) (Small, 2017; McPartland, 2018; Barcaccia et al., 2020). Ren et al. (2021), by means of an extensive analysis of genomes, recently demonstrated that current hemp and drug cultivars diverged from an ancestral gene pool, the extant members of which are feral plants and landraces in China, and thus comprise a single species, *C. sativa*. Regardless of ancestry, their descendants now occur throughout the world, especially in temperate locations favorable for their growth (Clarke and Merlin, 2013; Small, 2017). A separate species, *Cannabis ruderalis*, is now rare in nature (Clarke and Merlin, 2013). The existence of many traditional local selections and landraces greatly complicates an understanding of this plant's genetics and intrarelationships. In this paper, cannabis and hemp will be used interchangeably.

Hemp landraces, cultivars, selections and accessions have been grouped by several authors using putative species (*C. sativa* vs. *C. indica*), leaf geometry (narrow leaf vs. wide leaf), amount of resin produced, ratio of (−)-trans-Δ9-tetrahydrocannabinol (THC) to cannabidiol (CBD), and typical use (fiber, seed, drug or a mixture of the three) (Meijer and Keizer, 1996; Hillig, 2005; Clarke and Merlin, 2016; Lynch et al., 2016). Small (2017) grouped all forms of *C. sativa/indica* into four categories: fiber, oilseed crop, psychoactive and medicinal sources, and “wild” escapes from cultivation. With so little information on hemp nematodes yet available, we will focus, where possible, on identification of cultivars and lines as high THC-low CBD (THC-dominant), high CBD-low THC (CBD-dominant), or low THC-low CBD categories. This last consists of varieties used primarily for fiber.

The goal of this paper is to provide an historically oriented, comprehensive review of the literature on nematode–hemp interactions.

## Materials and methods

### Hemp literature

Articles were gathered from the Web of Science database using the terms “Cannabis × nemato*” and “hemp × nemato*”. The abstract of each article was read to determine if its subject matter concerned plant-parasitic nematodes and *Cannabis sativa*, as many hits dealt with sunn hemp (*Crotalaria juncea* L.), Bombay hemp or kenaf (*Hibiscus cannabinus* L.) and other plants. To determine the total number of records for root-knot nematodes, the terms “Meloidogyne”, “Heterodera radicicola” and “Heterodera marioni” were searched. Because Web of Science contains a number of duplicate figure and data table entries when the “all databases” feature is selected, the CABI citation database was similarly searched to provide possibly more compact results. Separate Web of Science and CABI citation database searches were conducted with the terms, “Cannabis, root, × chem*”. Compounds and types of compounds were also searched with the term nematod* (e.g., “phytosterols × “nematod”; “β-sitosterol × nematod*”.

## Results

The available literature on hemp nematodes is rife with reference to compiled lists and books that repeat previous records. We have emphasized primary literature rather than secondary or tertiary literature. For instance, Richter (1911) is often cited as the first report of root-knot nematode on hemp, but he properly attributed the record to Passon (1909), which was located for confirmation. In a more complicated case, Goodey et al. (1965) listed *Ditylenchus dipsaci* as a parasite of hemp, citing Steiner and Buhrer (1932). However, the latter authors listed the combination without specifying a source, although they did write that their list was for U.S. records. We have been unable to find a U.S. report from which Steiner and Buhrer could have extracted the record; it is possible that this earliest record is buried in a state agricultural annual report, or might even have been an unpublished remark made to Steiner or Buhrer. The first unequivocal U.S. record we have found for any nematode on *C. sativa* is that of Whittle and Drain (1935), for root-knot nematode on hemp in Tennessee. Most earlier reports did not mention specific cultivars or lines, which makes them of limited value in understanding host-parasite relationships. The present literature search yielded 38 primary citations of nematodes as parasites of hemp and 23 citations on uses of hemp for management of nematodes. Based on the very limited literature, the most important genera occurring on hemp are *Meloidogyne* (root-knot nematodes) and *Pratylenchus* (lesion nematodes); *Ditylenchus* (stem nematode) also has been mentioned frequently (see McPartland et al., 2000) but most reports are derivative and do not add knowledge (e.g., Ceapoiu, 1958; Dempsey, 1975). McPartland et al. (2000) gave an excellent general overview of plant-parasitic nematodes and discussed the nematodes reported on hemp up to that time, including management alternatives and Wang (2021) provided a good section on nematode symptoms and diagnosis.

## Nematodes parasitizing hemp

### Root-knot nematodes (Meloidogyne spp.)

**Figure 1:**
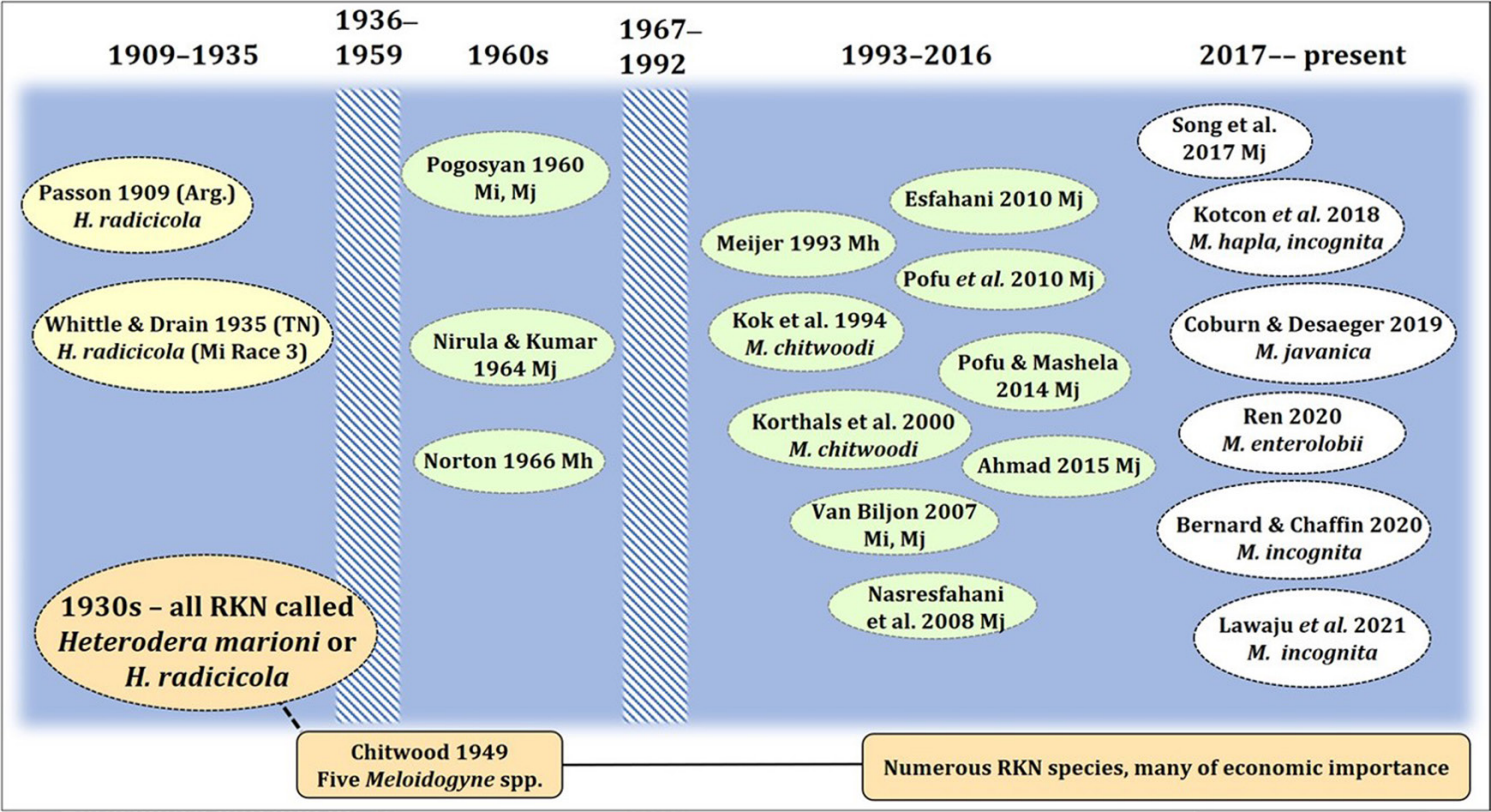
Time chart of primary literature reporting root-knot nematode-*Cannabis sativa* associations. Mh, *Meloidogyne hapla*; Mi, *M. incognita*; Mj, *M. javanica*.

Parasitism of hemp by species of *Meloidogyne*, the root-knot nematodes (RKN), is well-established, although based on just a handful of papers. The Web of Science and CABI databases contain 40,355 and 29,679 records, respectively, of root-knot nematode publications as of 16 August 2021, but only 20 deal with specific root-knot nematodes on hemp (Fig. 1). Of the many described species of *Meloidogyne*, five have been examined at least cursorily for their ability to reproduce on hemp: *M. chitwoodi, M. enterolobii*, *M. hapla*, *M. incognita* and *M. javanica*. All are cosmopolitan except *M. chitwoodi*, which nevertheless is widespread in North America and Europe. Many other economically significant root-knot species still must be checked to determine if they can parasitize hemp. Early reports (pre-1949) of root-knot nematodes identified them as *Heterodera marioni* or as *H. radicicola*, taxa that included all root-knot nematodes. Passon (1909) reported *H. radicicola* on hemp in Brazil, but the particular nematode species is unknowable. Buhrer et al. (1933) listed *Cannabis sativa* as a host of *Heterodera marioni* but did not cite the source of that record. The first definitive U.S. report of root-knot nematode (as *H. radicicola*) on hemp was that by Whittle and Drain (1935), who listed hemp as slightly infested. Mateeva (1995) reported hemp to be resistant to *Meloidogyne* spp. relative to tomato and cucumber but did not indicate a particular nematode species nor the hemp source. The susceptibility of plants to root-knot nematodes and other parasitic nematodes, especially in controlled greenhouse studies, is often calculated as Rf = Pf/Pi, where Rf is the reproductive factor, Pf is the final number of nematodes or eggs, and Pi is the initial inoculum. However, very few papers have reported Rf values or provided enough information to calculate this value.

**Figure 2:**
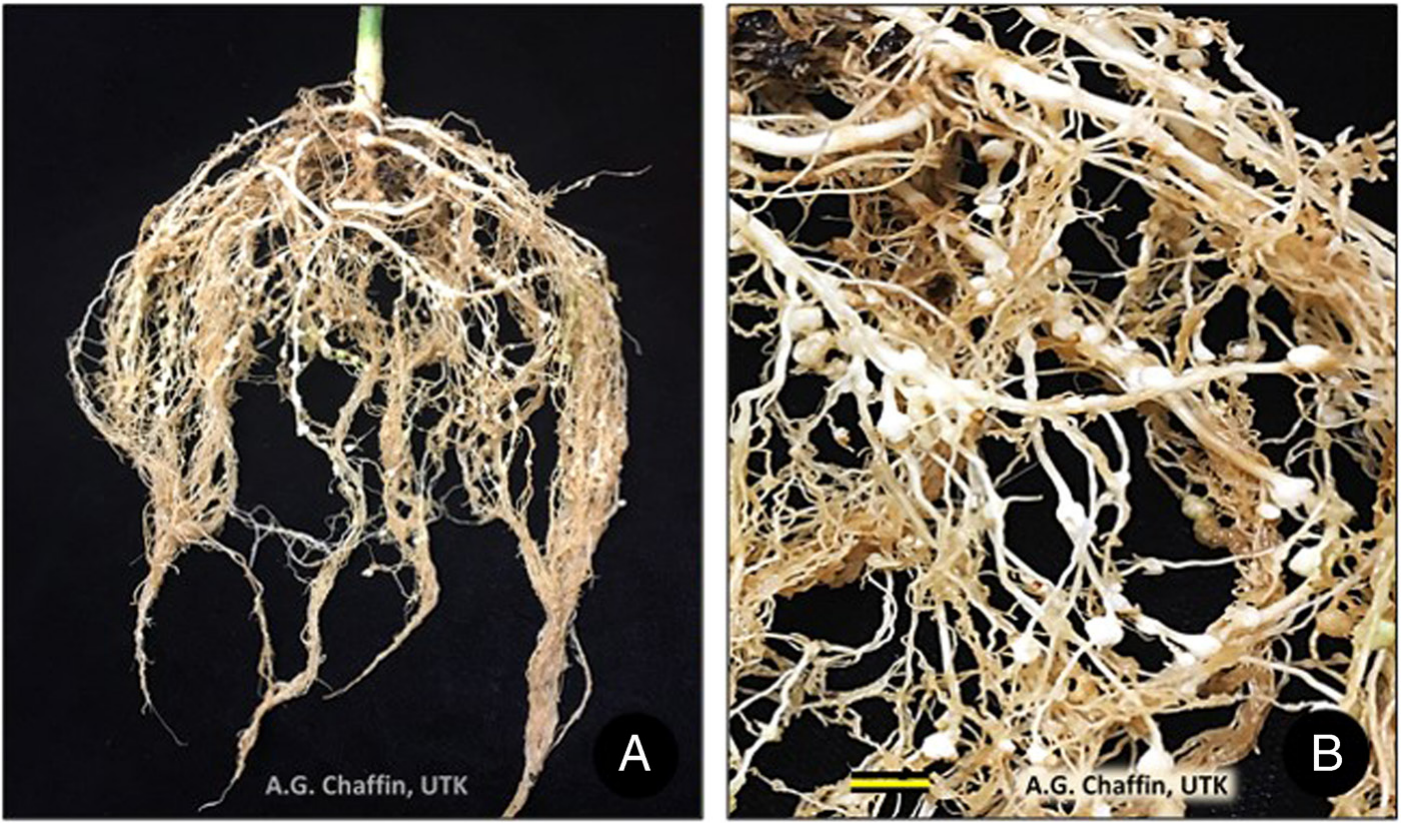
Galling of *Meloidogyne incognita* on *Cannabis sativa* ‘Cherry’, Pi = 10,000 eggs. (A) Entire root system. (B) Closeup of small, white galls. Scale = 5 mm.

Galls induced on hemp roots by *M. hapla*, *M. incognita* and *M. javanica* are small, whitish and hard (Fig. 2), not exceeding about 4 mm in diameter (Kotcon et al., 2018; Coburn and Desaeger, 2019; Bernard and Chaffin, 2020). Both small and large galls on hemp were formed by *M. enterolobii* (Ren et al., 2020) but gall dimensions were not given.

### *Meloidogyne chitwoodi* Golden, O’Bannon, Santo and & Finley (Columbia root-knot nematode)

Korthals et al. (2000) reported that hemp was not a host for *M. chitwoodi* based on the nematode's failure to increase in hemp field plots. This nematode had very limited reproduction on fiber cv. ‘Kompolti Hibrid TC’ and the Rf was well below replacement level (Kok et al., 1994).

### *Meloidogyne enterolobii* Yang and Eisenback (Pacara earpod tree root-knot nematode, guava root-knot nematode)

This rather recently described species is now considered a cosmopolitan, economically important nematode with a broad host range (Castillo and Castagnone-Sereno, 2020). *Meloidogyne enterolobii* induced many galls on infested industrial hemp plants in the field and on the industrial cv. Longma No. 5 in greenhouse tests (Ren et al., 2020); these authors considered *M. enterolobii* to be a major potential threat to hemp production. The average Rf in the greenhouse experiments was 18.2.

### *Meloidogyne hapla* Chitwood (northern root-knot nematode)

*Meloidogyne hapla* is the most common species in the northern U.S., and thus the common name, but it is actually cosmopolitan (CABI/EPPO, 2002). It was first reported as a parasite of hemp by Norton (1966), who found a moderate gall rating of 1.9 (0–4 scale) on seed-grown plants of unreported provenance. Root galling and egg mass production of *M. hapla* on 123 hemp accessions varied widely (Meijer, 1993); in particular, fiber accessions differed significantly in degree of infection and reproduction. Kotcon et al. (2018) found root galling on five industrial hemp cultivars (‘Canda’, ‘Delores’, ‘Fedora’, ‘Felina 32’, ‘Futura 75’) in a greenhouse experiment. Galling was higher on ‘Felina 32’ than on the other four cultivars, but reproduction did not differ.

### *Meloidogyne incognita* (Kofoid and White) (southern root-knot nematode)

This nematode also is cosmopolitan and probably accounts for more crop loss worldwide than any other species. Despite the importance of *M. incognita* in agriculture, its pathogenicity on hemp has hardly been examined. Whittle and Drain (1935) were the first to experimentally study this species on hemp, as *Heterodera radicicola.* Cotton was a good host, but not tobacco or peanut, which identifies this population as *M. incognita* Race 3 (Taylor and Sasser, 1977). Pogosyan (1960) and Nirula and Kumar (1964) reported *M. incognita* on *C. sativa* in Armenia and India, respectively, but did not provide details on hemp type or symptoms. In a study by Kotcon et al. (2018), the fiber cv. ‘Canda’ supported much greater galling and reproduction than did ‘Felina 32’. In recent greenhouse experiments the fiber type ‘Delores’ was a very compatible host for *M. incognita* Race 3, with numerous galls, egg masses, and high reproduction (up to 1 million eggs/root system) (Bernard and Chaffin, 2020; Hansen et al., 2020) (Fig. 1). The reproductive factor (RF) was 36–81. A CBD-dominant cultivar, ‘Wife’, had only a few galls and minor reproduction (Rf = 0.2) (Bernard and Chaffin, 2020). However, the CBD-dominant cultivar ‘Charlotte's Web’ had a high Rf value (39.6) and other CBD-dominant cultivars ranged from 2.4 to 17.5. Van Biljon (2017) found that most of 10 tested cultivars were tolerant or susceptible to *M. incognita* Races 2 and 4, but that three of the 10 were resistant to Race 2, including ‘Futura 75’. This cultivar was a good host for M. incognita Race 3 (Bernard and Chaffin, 2020). Clearly there is a wide variation in susceptibility of hemp to *M. incognita*, both by cultivar and by nematode isolate, which could provide a useful tool for management of this nematode.

Evidence is beginning to appear that *M. incognita* is a threat to field production of hemp crops. This nematode caused galls on industrial hemp in a North Carolina production field, and although the infection was light it was associated with plant stunting (Thiessen et al., 2020). In Alabama, growth of cultivars ‘Boax’ and ‘Otto2’ was reduced and root systems exhibited galling demonstrated to be caused by *M. incognita* Race 3. In a subsequent greenhouse test this nematode isolate successfully caused galls and reproduced on ‘Maverick’ hemp, a CBD-dominant cultivar, with an Rf of 2.1 (Lawaju et al., 2021).

The reproduction factor (Rf) is a useful means of estimating the suitability of a host for nematode increase, but comparisons from one study to another must be made cautiously. In the Alabama study of Lawaju et al. (2021), 500-cm^3^ pots were used, with an initial inoculum (Pi) of 2,500 eggs; the experiment was run for 45 days and resulted in an Rf of 2.1 on ‘Maverick’. In contrast, Bernard and Chaffin (2020) used 1000-cm^3^ pots with a Pi of 5,000 eggs and ran their experiments for 60 days, with an average Rf on ‘Delores’ of 45 and on ‘Wife’ of 0.2. Several other factors prevent a close comparison of the two protocols. The two nematode isolates could have differing virulence on hemp and *a priori* knowledge of a hemp cultivar's susceptibility is necessarily unknown, making Rf comparisons difficult. Comparative and cooperative studies are needed to better understand the complexity of root-knot nematode–hemp interactions.

### *Meloidogyne javanica* (Treub) (Javanese root-knot nematode)

This nematode reproduced successfully on four tested fiber cultivars in South Africa, but the percent population increase differed among the cultivars: increase was highest on ‘Kompolti’ and ‘Ferimon’, intermediate on ‘Felina 34’ and lowest on ‘Futura 75’ (Pofu et al., 2010). Root and shoot weights of all cultivars were unaffected by nematode infection. Pofu and Mashela (2014) found the rate at which *M. javanica* increased on hemp cultivars to vary markedly, with optimal numbers occurring on ‘Kompolti’, ‘Futura-75’ and ‘Ferimon’ at 56 days, while optimization on ‘Felina-34’ required 151 days. Unspecified varieties of *C. sativa* were hosts for *M. javanica* in India (Nirula and Kumar, 1964) and Iran (Esfahani and Ahmadi, 2010), and the nematode caused moderate galling on hemp growing as weeds in winter tomato fields in Pakistan (Ahmad et al., 2015). In Florida, six industrial hemp cultivars and land-races (‘Helena’, ‘Tygra’, ‘Fibranova’, ‘Eletta Campana’, ‘Carmagnola’, and ‘Carmagnola Selezionata’) were tested in the greenhouse for susceptibility to *M. javanica* (Coburn and Desaeger, 2019). All six cultivars were excellent hosts, with Rfs of 34–52, but plant biomass was not affected. Galls were non-coalesced, small, and numerous throughout the entire root system.

### Cyst nematodes (*Globodera* and *Heterodera* spp.)

Cyst nematodes are sedentary endoparasites. They typically have smaller host ranges than the common root-knot nematodes. Several species are among the most serious parasites of their favored host plants, including *G. pallidum* and *G. rostochiensis* (potato), *H. glycines* (soybean) and *H. schachtii* (sugar beet). *Cannabis sativa* is not a favored host for any known cyst nematode species, and *Globodera* spp. have never been reported from the plant.

### *Heterodera humuli* Filipjev (hop cyst nematode)

Summaries and illustrations of *H. humuli* include those of Stone and Rowe (1977) and Spaar et al. (1990). This nematode, described in 1934, had previously been considered *H. schachtii* “hops strain” (Filipjev and Schuurmans Stekhoven, 1941). Hollrung (1890) reported *H. schachtii* on hemp as well as many other plants and later found *H. schachtii* juveniles on hemp roots (Hollrung, 1891). These records certainly indicate a wide range of other later-described cyst nematode species. Goodey et al. (1965), in their list of hosts of plant-parasitic nematodes, suggested that Hollrung's record might have been *H. humuli*. Franklin (1951) indicated that *H. humuli* reproduced successfully on hemp, but did not provide additional information. In a laboratory study *H. humuli* successfully matured on hemp (Winslow, 1954). Jensen et al. (1962) considered hemp to be a host of *H. humuli* on the basis of previous European records, although not from experimental results. Conversely, hemp-root diffusates did not stimulate *H. humuli* egg hatch (De Grisse and Gillard, 1963), and isolates of this nematode from hop and nettle did not develop on *C. sativa* (Subbotin, 1986). None of these reports specified the hemp type or cultivar. As the identifications of the *H. humuli* populations in these latter experiments were made by experienced nematologists it seems likely that its ability to parasitize hemp is variable; the nematode may have host-selected populations or hemp varieties may differ in reaction as they do with root-knot nematodes.

### *Heterodera glycines* Ichinohe (soybean cyst nematode)

Densities of *H. glycines* declined in a hemp-soybean rotation (Zhang et al., 2013), indicating that hemp could be used for *H. glycines* management. Rotation with nonhosts is a recommended management option for controlling this nematode (Schmitt et al., 2004).

### *Heterodera ripae* Subbotin, Sturhan, Waeyenberge and Moens

This nematode was collected from common nettle (*Urtica dioica* L.) in Spain. Experimental tests demonstrated that neither hemp nor hop was a host for *H. ripae* (López-Robles et al., 2011).

### *Rotylenchulus* spp. (reniform nematodes)

*Rotylenchulus reniformis* (Linford and Oliveira) is the most economically important member of its genus, distributed worldwide in tropical and subtropical regions. In the southeastern U.S. it is a serious pathogen of cotton, soybean and other crops, but has not yet been studied specifically on hemp. In South Africa, Dippenaar et al. (1996) found reniform nematodes (species not identified) as a natural constituent in the field soil of their experiments but did not state whether they parasitized hemp.

### Lesion nematodes (*Pratylenchus* spp.)

Lesion nematodes are migratory endoparasitic nematodes. These nematodes enter roots and move through the cortex, killing the cells as they feed and weakening plant defenses. Romaniko (1963) reported *Pratylenchus globulicola* (Romaniko) on hemp. This species was synonymized with *P. penetrans* (Cobb) by Loof (1978), but was later validated as a distinct species (Ryss et al., 2015). Fiber hemp ‘Kompolti Hibrid TC’ was an excellent host of *Pratylenchus penetrans* (Kok et al., 1994), with numbers comparable to a standard susceptible host; in a subsequent experiment on an unnamed fiber cultivar *P. penetrans* increased its numbers 4–6 × above the initial inoculum (Kok and Coenen, 1996). Dippenaar et al. (1996) found similar results on ‘Kompolti’ in South Africa, but other fiber cultivars (‘Fedora-19’, ‘Futura-77’, ‘Felina-34’, ‘Secuini’) were resistant. Also in South Africa, Van Biljon (2007a, 2007b) reported *P. teres*, *P. zeae* and *P. scribneri* associated with hemp; yield of ‘Kompolti’ fiber hemp was increased in lesion nematode-infested fields treated with 6 metric tons of chicken litter/hectare. In a Dutch field study, *P. penetrans* reproduced better on hemp than on 18 other field, vegetable, and forage crops (Brommer and Beers, 2001). *Pratylenchus thornei* did not invade hemp roots in a Syrian field experiment (Greco et al., 1988). There clearly is significant need for more research on hemp-*Pratylenchus* relationships. Members of this genus often partner with several wilt fungi in a number of destructive disease complexes, but the potential for nematode-wilt complexes on hemp has not yet been studied (see section below).

### *Ditylenchus dipsaci* (Kühn) (stem nematode) and relatives

This nematode and its host-parasite relationships have been thoroughly reviewed by Sturhan et al. (2008). *Ditylenchus dipsaci* invades stems of susceptible plants and causes maceration of cortical and pith cells as they feed by secretion of pectinases and other enzymes (Riedel and Mai, 1971). Mating and reproduction take place inside the stem. Symptoms result in collapse and lodging of affected plants. Kirchner (1906) provided the first description of *D. dipsaci* infection of hemp, followed soon by Peglion (1901, as *Tylenchus devastatrix*); McPartland et al. (2000) provided a list of later European records. This species has many host races (Dropkin, 1988; Ladygina, 1988; Sturhan and Brzeski, 1991) and an enormous host range, but is most serious on alfalfa (lucerne), onion and a wide range of floral bulbs; historically it also was the most damaging parasite of teasel. Steiner and Buhrer (1932) listed *C. sativa* as a host for *D. dipsaci*, but with a question mark and without citing a source. Symptoms of *D. dipsaci* infestation on hemp have been described and(or) illustrated by Kirchner (1906), Peglion (1901), Kotthoff (1937), Mezzetti (1951), Ferri (1961) and Rak Cizej and Poličnik (2018), and are similar to those on crops such as alfalfa (Sturhan and Brzeski, 1991). Peglion (1901), summarized by Schumann (1903) and cited in Marcinowski (1909), determined that hemp planted as seed was susceptible to *D. dipsaci*, but older plants were not infected when nematode-rich soil was spread around them. Sturhan and Brzeski (1991) stated that *D. dipsaci* caused significant damage to hemp in Turkey, although they did not provide attribution or additional details for the statement.

Stem nematodes on hemp were placed in the rye race by Kotthoff (1950) but in the flax-hemp race by Kir’yanova and Krall’ (1971). Skarbilovich (1951) found slight infection of hemp by the potato race and moderate infection by the onion race. Ladygina (1988) and Janssen (1994) did not mention the flax-hemp race, nor did they list hemp or flax in any other race; however, Sturhan and Brzeski (1991) thought that the flax-hemp and rye races were identical. *Ditylenchus dipsaci* is now recognized as a species complex (Subbotin et al., 2005), with several named and economically significant species (Saadi et al., 2019). The taxonomic status of *D. dipsaci* populations capable of parasitizing *C. sativa* has not been studied, but the various results and placements suggest that the relationship of the nematode and *C. sativa* may involve cryptic members of the *D. dipsaci* complex.

### Ectoparasitic nematodes

Ectoparasites live in soil and feed on epidermal and cortical cells typically without entering the root. Most habitats contain multiple species of these nematodes. This taxonomically diverse group includes genera such as stunt nematodes (*Tylenchorhynchus* and relatives), spiral nematodes (*Helicotylenchus, Rotylenchus, Scutellonema*), ring nematodes (*Mesocriconema* and relatives) and dagger nematodes (*Xiphinema* spp.). Very little attention has been paid to ectoparasitic nematodes on hemp. A number of associations with hemp have been reported based mainly on field samples, but actual feeding or maintenance of nematodes on hemp roots has not been conclusively established. Nematode communities in a Russian hemp soil consisted of 21 species; of the seven plant-parasitic species, *Tylenchorhynchus dubius* was the most common, comprising 38% of collected nematodes, followed by the migratory endoparasite *Pratylenchus penetrans* (13%) (Skarbilovich, 1969). Sturhan (1963) collected a needle nematode, *Longidorus maximus*, in the rhizosphere of hemp but could not definitively determine that root symptoms were caused by this nematode. In South Africa, at least 25 species of nematodes, most of them ectoparasites, have been collected from hemp soils (summarized in Van Biljon, 2017).

### Disease complexes

A disease complex is the result of “…a synergistic interaction between two organisms” (Back et al., 2002). Typically, this interaction results in much more serious disease than either pathogen would cause by itself. These interactions have been categorized as utilization of nematode-induced wounds by soil-borne pathogens, nematode-induced physiological changes to the host plant, modifications within the rhizosphere, reduction of host resistance, and pathogen-induced changes to the host plant (Back et al., 2002). Nematodes most frequently form disease complexes with species of the fungal genera *Fusarium* and *Verticillium*. Environmental factors also make a contribution to the severity of disease complexes. Nyczepir et al. (1997) summarized the roles that the ring nematode *Mesocriconema xenoplax*, bacterium *Pseudomonas syringae* pv. *syringae* and soil pH play in the severity of the peach tree short life complex. An advanced computer analysis of possible factors in Verticillium wilt of mint (Wheeler et al., 2019) identified four important predictors of wilt. The most important factor was the lesion nematode *Pratylenchus penetrans*, followed by crop age, cultivar and the fungus *Verticillium dahliae*.

Hemp disease complexes have not been reported as fact, although Ferraris (1935) suggested an association of *D. dipsaci* with the downy mildew *Pseudoperonospora cannabina*. Hemp is a host for several fungi known to partner with nematodes in disease complexes, especially *Fusarium oxysporum* and *Verticillium dahliae* (Spaar et al., 1990; McPartland et al., 2000; Gwinn et al., 2022; Wang, 2021). However, no research has been conducted on nematode-fungal pathogen disease complexes on hemp.

## Hemp amendments and plant products for nematode management

Much of the research reported on hemp concerns its use in various forms as a soil amendment (Table 1). This approach deserves more research, as increasing hemp production should lead to more crop residue that can be incorporated back into the soil to increase organic matter and possibly stimulate natural enemies of soil-borne plant pathogens. McPartland (1997) provides a good discussion of hemp amendments and their potential for pest and pathogen management. However, contradictory experimental results are common. For instance, several authors reported high *M. incognita* J2 mortality when exposed to *C. sativa* leaf extracts (Mukhtar et al., 2013; Saxena and Gupta, 2004), while others have found no effect (Nazar and Nath, 1989). The fact that hemp leaves in various preparations appear to have efficacy against many plant nematodes (Table 1) suggests a rich leaf chemistry, separate from the inflorescences, that could help with organic nematode management. However, these compounds have not yet been investigated as separate entities for biocontrol activity.

**Table 1:** Experimental results on the use of hemp tissues and extracts for nematode management.

**Treatment with hemp**	**Target nematode(s)**	**Results**	**Authors**
Rotation crop with soybean	*Heterodera glycines*	J2 numbers were reduced 30%, soybean yields increased 11% when soybean followed hemp	Zhang et al. (2013)
Chopped leaves incorporated into potted soil	*Meloidogyne incognita*	Reproduction on tomato reduced 74% but plant growth parameters not significantly improved	Khanna and Sharma (1998)
Chopped leaves	*Tylenchorhynchus* sp. *Helicotylenchus* sp.	42% reduction 82% reduction	Somvanshi and Gupta (2003)
Pulverized leaves incorporated into soil	*Meloidogyne incognita*	Galls decreased up to 54% on subsequent crop	Kayani et al. (2012)
Chopped leaves	*Meloidogyne incognita*	Incorporation of leaves did not improve growth of False Eranthemum infected with the nematode	Ganaie and Khan (2017)
Whole plant extract	*Hoplolaimus indicus Longidorus* sp. *Pratylenchus* sp. *Xiphinema americanum*	High nematode mortality	Pandey and Dwivedi (2000)
Plant extract	*Aphelenchoides composticola*	70% mortality after 72 hr	Khanna et al. (1988) ^a^
Green manure or plant powder	*Meloidogyne incognita Helicotylenchus dihystera Tylenchorhynchus nudus Pratylenchus zeae*	For green manure, increased plant growth, reduced nematode numbers; for plant powder, reduced nematode numbers but no improvement in plant growth	Thakur (2014)
Aqueous extract of stems, leaves, inflorescences	*Strongylus papillosus Haemonchus contortus* (mammal parasites)	Weak effect on *S. papillosu*s J1, J2; no effect on J3 or *H. contortus*	Boyko and Brygadyrenko (2019)
Ethanol extracts of roots, leaves, inflorescences	*Steinernema* spp. *Heterorhabditis bacteriophora* (EPNs)	Highest attraction of dauer juveniles was to inflorescence extracts	Laznik et al. (2020)
Water extracts of plant tissue	*Meloidogyne incognita*	High mortality of J2	Singh and Singh (2002)
Root and shoot extracts	*Hoplolaimus indicus Rotylenchulus reniformis Tylenchorynchus brassicae*	Effective mortality of nematode species tested down to 1:10 dilution	Haseeb et al. (1978)
Root extract	*Meloidogyne incognita*	Inhibited egg hatch, increased juvenile mortality	Ganaie and Khan (2016)
Leaf extract	*Meloidogyne incognita*	74–96% juvenile mortality	Saxena and Gupta (2004)
Leaf extract	*Meloidogyne incognita*	88% inhibition of egg hatch	Adegbite (2011)
Water extracts from ground leaves	*Meloidogyne javanica*	J2 mortality was 92% or higher at dilutions of 1:5 to 1:40	Nandal and Bhatti (1983)
Water extracts from ground leaves	*Meloidogyne javanica*	Activity of 10-day-old extracts to J2 remained above 94%	Nandal and Bhatti (1986)
Hot and cold-water leaf extracts	*Aphelenchoides composticola*	Mortality increased with concentration, hot-water extracts more effective than cold extracts	Grewal (1989)
Aqueous leaf extract	*Heterodera cajani*	100% mortality of J2 after 24 and 48 hr of exposure	Mojumdar et al. (1989)
Boiled aqueous leaf extract	*Meloidogyne incognita*	Soaking chickpea seed in extract totally inhibited J2 invasion of seedlings	Mojumder and Mishra (1991)
Water extract of leaves	*Meloidogyne incognita*	100% mortality of juveniles	Sharma (1996)
Water extracts of macerated leaves	*Meloidogyne incognita*	Plant growth improved and J2 numbers reduced 40% on brinjal (eggplant) but not as much as with margosa extract	Shah et al. (2018)
Methanol extracts of dried, pulverized leaves	*Meloidogyne javanica*	89% mortality of J2 in 5% solution	Amini et al. (2011)
Water extracts from ground leaves	*Meloidogyne incognita*	Extracts gave high mortality of J2 after 72 hrs	Mukhtar et al. (2013)

a Data from summary, original paper not seen.

## Nematodes and *Cannabis sativa* secondary metabolites

**Figure 3:**
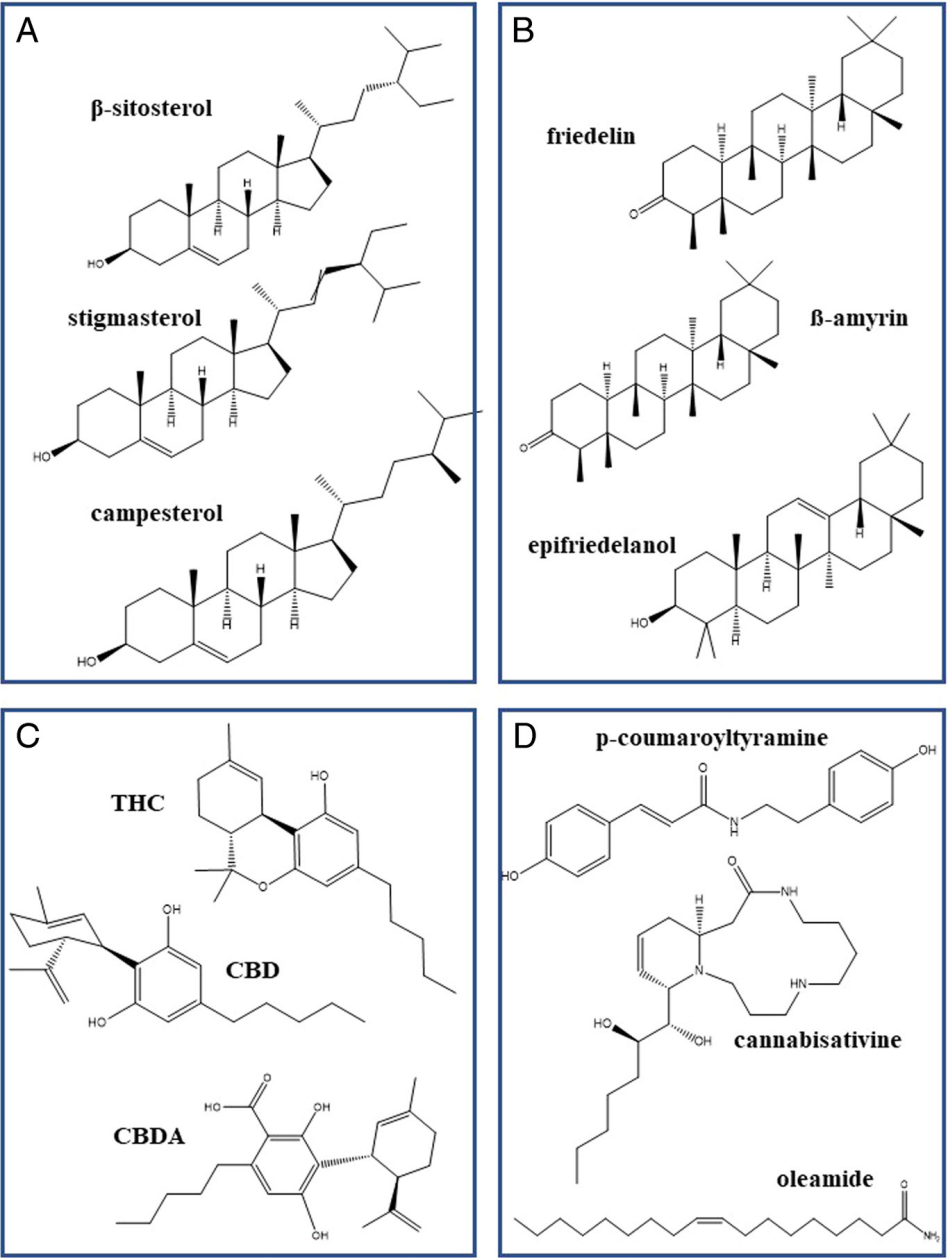
Chemicals isolated from *Cannabis sativa* roots. (A) Phytosterols. (B) Triterpenoids. (C) Cannabinoids: - (−)-trans-Δ9-tetrahydrocannabinol (THC); cannabidiol (CBD); cannabidiolic acid (CBDA). (D) Nitrogen-containing compounds.

Inflorescences, leaves, and roots of *Cannabis sativa* contain a wealth of secondary metabolites that are bioactive and have potential as sources of biopesticides (Andre et al., 2016). Cultivars of medicinal *C. sativa*, CBD-dominant and THC-dominant chemotypes, respectively, are cultivated primarily for the medicinal cannabinoids in the inflorescences (approximately 20% by weight); inflorescences also contain terpenoids and flavonoids but at lower concentrations. Leaves contain less than 10% of the cannabinoids and terpenoids in inflorescences but have higher concentrations of flavonoids (Jin et al., 2020). In general, extracts from inflorescences had greater bioactivity against entomopathogenic nematodes than those from leaves or stems. Extracts of leaves from one THC-dominant chemotype and one CBD-dominant chemotype were attractive to *Steinernema feltiae* and *S. carpocapsae*, respectively, whereas extracts from another THC-dominant chemotype and another CBD-dominant chemotype were weak repellents to *S. carpocapsae* (Laznik et al., 2020). Sterols and triterpenes are the primary components of stem barks and roots (Jin et al., 2020), but other compounds such as aliphatics (Kornpointner et al., 2021), amides (Yamamoto et al., 1991), and cannabinoids (Gul et al., 2018) have also been isolated from roots (Fig. 3). Numerous root compounds impact nematode behavior and development and may influence many of the host-nematode interactions described above.

### Phytosterols

Phytosterols (24 C sterols) (Fig. 3A) were among the first compounds to be described from roots of *C. sativa* (Slatkin et al., 1975; Sethi et al., 1977). These compounds and triterpenes are the predominant compounds isolated from *C. sativa* roots, and β-sitosterol is the sterol found in greatest concentration (Jin et al., 2020). Total sterols (β-sitosterol + campesterol + stigmasterol) were greater in THC-dominant strains than in an intermediate strain (TCH levels lower and CBD levels higher than in the THC-dominant). Only stigmasterol was not greater in THC-dominant strains (Jin et al., 2020, 2021). β-sitosterol content of roots of a strain with THC levels intermediate to the THC- and CBD-dominant cultivars was higher when plants were grown in aeroponic culture than those grown in soil, but campsterol and stigmasterol were similar among cultivation methods. Plants grown in aeroponics, however, had greater root mass so total content/plant was 20 times higher for β-sitosterol and 10-fold higher for the other phytosterols (Ferrini et al., 2021). In addition to those described above, two new phytosterols (stigmastanol and fucosterol), three steroid ketones, and two steroid hydrocarbons were isolated from roots of field-grown CBD-dominant strains grown for fiber; this was a first report of steroid hydrocarbons in *C. sativa* (Kornpointner et al., 2021). A glycoside, β-sitosterol-β-D-glucoside, was reported in a CBD-dominant strain (Elhendawy et al., 2018).

Sterols are essential nutrients for nematodes (Chitwood and Lusby, 1991), and nematode infection can alter phytosterol composition of plant roots. Infection and colonization of tomato by *M. incognita* juveniles resulted in downregulation of the sterol 22C-desaturase gene and subsequent increased ratios of β-sitosterol/stigmasterol (Cabianca et al., 2021). Amounts of sterols and their distribution in the plant were not different in two near-isogenic line of cotton that were susceptible or resistant to *M. incognita* (Hedin et al., 1995). Treatment of newly hatched juveniles of *M. incognita* with β-sitosterol resulted in only 26.1% mortality after 72 hr (Li and Xu, 2012). Exposure of *Heterodera zeae* to 1% β-sitosterol or stigmasterol resulted in mortalities of 100 and 90%, respectively, but fractions of extracts from *Tagetes patula* containing β-sitosterol glucoside did not cause mortality (Bano et al., 2019). Stigmasterol (100 μg/mL) was nematicidal to 94.5% of juveniles of *Nacobbus aberrans*, whereas at the same concentration, β-sitosterol treatment resulted in immobilization of 68.7%. (Velasco-Azorsa et al., 2021). In an assay used to predict anti-nematodal activity of compounds, stigmasterol was not active as a nonpeptide agonist ligands for nematodal G-protein-coupled receptors (Fotso et al., 2014). Nematode parasites of mammals may be more sensitive to phytosterols; *Ascaris suum* individuals curled immediately on exposure to β-sitosterol and were paralyzed (Villaseñor et al., 2002).

### Terpenes

Although carvone and dihydrocarvone were reported in *C. sativa* weeds collected by Sethi et al. (1977), neither they nor the other terpenes reported from inflorescences and leaves (mono- and sesquiterpenes) have been reported in roots in recent literature. Roots contained triterpenoids, the two primary triterpenoids (friedelin and epifriedelanol) and ß-amyrin, (Fig. 3B) all of which were higher in the intermediate cultivar than in the THC-dominant (Jin et al., 2020). The first two compounds were the first reported from roots of a THC-dominant chemotype (Slatkin et al., 1971) and have subsequently been reported in *C. sativa* weed populations (Sethi et al., 1977), fiber (CBD-dominant) hemp (Kornpointner et al., 2021) and medicinal cannabis (Elhendawy et al., 2018; Jin et al., 2020, 2021). Glutinol and amyrone were also isolated from the fiber hemp (Kornpointner et al., 2021). In a study on impact of plant harvest date and drying on concentrations of the two primary triterpenoids in roots of fiber ‘Futura’ grown in Austria, there were no differences in friedelin content of roots harvested in July and August and air-dried, but epifriedelanol levels were lower in July. Harvesting roots in October and drying at 45 C resulted in lower levels of both compounds (Kornpointner et al., 2021).

Carvone treatment inhibited egg hatch inhibition and increased juvenile mortality in *M. incognita* (Goyal et al., 2021). There have been no reports on the effects of root-produced triterpenoids on plant-parasitic nematodes. β-Amyrin acted as an excellent antioxidant and reduced intracellular reactive oxygen in a wild type strain of *C. elegans* (N2). When juveniles of a transgenic strain (NL5901) (expresses YFP in muscles with human α-synuclein and is used to study Parkinson's Disease) were treated with β-amyrin, juveniles had reduced cellular damage and α-synuclein aggregation (Wei et al., 2017). Friedelin was not active as a nonpeptide agonist ligand for nematodal G-protein-coupled receptors (Fotso et al., 2014). Free triterpenes and triterpenic saponins have been proposed as bio-nematicides for both animal and plant parasites (reviewed in D’Addabbo et al., 2011; Isah et al., 2016). Solanoeclepin A was recently identified as a hatching stimulant for eggs of *Globodera rostochiensis* (Shimizu et al., 2020) and *G. pallida* (Guerrieri et al., 2021), and it is “extremely active in sub-nM concentrations” (Guerrieri et al., 2021). Isolated triterpenoids had less impact on egg hatch, motility and viability of *M. incognita* than plant extracts high in triterpenoids, suggesting that other compounds in the extracts increase activity of these compounds (Dube et al., 2019).

### Cannabinoids

Cannabinoids (Fig. 3C) are minor components of *C. sativa* roots (Jin et al., 2021; Kornpointner et al., 2021). Gul et al. (2018) isolated ten cannabinoids from the roots of three cultivars of *C. sativa*. ‘MX’, a THC-dominant cultivar, had the highest levels of total cannabinoids, and ‘V1-19’, a CBD-dominant cultivar, had the lowest levels of all ten cannabinoids, including CBD. In ‘MX’, CBD concentrations in the roots were 12.59 ppm, and Δ9-THC concentrations were 2.375 ppm. In all cultivars, cannabidiolic acid (CBDA) and Δ9-tetrahydrocannabinolic acid-A (THCAA) concentrations were present at the highest concentrations, and concentrations of these precursors were directly correlated to CBD and Δ9-THC, respectively. Concentrations of nine cannabinoids were higher in the mother plants of ‘MX’ and ‘V1-19’ than in any of three daughter plants; tetrahydrocannabivarian (THCV) was produced only in roots of one daughter plant. Cannabinoids were produced in hairy root cultures but only at low concentrations (below 2.0 μ g/g dry weight) (Farag and Kayser, 2015).

In the primary literature, there are no reports on the effects of cannabinoids on plant-parasitic nematodes, but they have been studied in *Caenorhabditis elegans.* Selected studies are reported here because even at low concentrations, endoparasitic species that have chronic exposure may be impacted that have chronic exposure may be impacted. [Although McPartland and Glass (2002) suggested that nematicidal effects of *C. sativa* were not related to cannabinoid receptors], several studies on alterations in behavior and physiology of *Caenorhabditis elegans* have been reported. Juveniles treated with cannabinoids exhibited a ‘dazed and confused’ behavior, with delayed responses to aversive stimuli, decreased feeding rates, slowed locomotion and increased unproductive turning” (Oakes, 2018); behavioral changes were modulated through complex signaling systems (Oakes et al., 2017, 2019). Cannabinoids can also affect nematode lifespan and health span of nematodes. Wang et al. (2020) examined the impact of CBD on two strains of *C. elegans*, N2 and a transgenic line used to study Alzheimer's Disease (BZ555); BZ555 expresses human amyloid-β (Aβ) protein throughout the nervous system. In the transgenic strain, juveniles had increased exploratory behavior, increased pharyngeal pumping in aged animals, and an improved chemotaxis response, but the in wildtype animals only exploratory behavior was affected.

### Nitrogen-containing compounds

Kornpointner et al. (2021) identified five aliphatic compounds in roots of a CBD-dominant cultivar grown for fiber, four of which were classified as putative artifacts. The fifth, oleamide in *C. sativa* roots, was isolated for the first time (Fig. 3D). Aliphatic compounds were also reported in roots of *C. sativa* ditchweed collected in Pakiston (Javaid et al., 2021) and a CBD-dominant cultivar (Elhendawy et al., 2018). Two additional classes of nitrogen-containing compounds have been reported in *C. sativa* roots: two amides (*p*-coumaroyltyramine and feruloyltyramine (Yamamoto et al., 1991; Elhendawy et al., 2018; Menezes et al., 2021) and two spermidine alkaloids (anhydrocannabisativine and cannabisativine) (Lotter et al., 1975; Turner et al., 1976; Elsohly et al., 1978; Menezes et al., 2021) (Fig. 3D). Activity of these compounds against nematodes has not been reported.

## Discussion

The evolution of modern nematology began in the mid-19th century, with the recognition of root-knot and cyst nematodes as possible detriments to crop production. In particular, the discovery of *Heterodera schachtii* (sugar beet cyst nematode, SBCN) as a major impediment to sugar beet production led to the first surveys of other crops to determine the host range of this nematode. By the time *H. schachtii* was recognized, hemp had long been cultivated around the world on all habitable continents, primarily for fiber production. Plant nematology research on many crops burgeoned during the widespread suppression of *C. sativa* production in the 20th Century, making legal *C. sativa* virtually a new crop in the past 20 years. There is no reason to expect that *C. sativa* will avoid its share of nematode-induced disease and disease complexes. Hemp-specific information on nematode management is almost non-existent, no doubt due to the very recent renascence in its large-scale cultivation and the general lack of reported damage to the crop. Nematicides used experimentally on hemp in South Africa reduced nematode numbers and increased yields (summarized in Van Biljon, 2017). As conventional nematicides need to be avoided in psychoactive and medicinal *Cannabis* production, other approaches will be necessary, such as plant resistance, cultural control, and effective biopesticides. Most fiber and CBD-dominant cultivars tested so far support moderate to extensive galling by root-knot nematodes (Kotcon et al., 2018; Coburn and Desaeger, 2019; Bernard and Chaffin, 2020; Lawaju et al., 2021), but resistance does exist within the species. The ability of *M. hapla* to reproduce on 123 hemp selections varied widely, even among fiber cultivars (Meijer, 1993). Fiber hemp cultivars ‘Futura 75’, ‘Diana’ and ‘Kubanskaia Rannaja’ were resistant to Race 2 of *M. incognita*, and several cultivars were tolerant of *M. javanica* infection (Pofu et al., 2010). Van Biljon (2017) considered that the nematode reproduction factors in those studies were still too high to use the tolerant cultivars for sustainable crop production. Bernard and Chaffin (2020) found the fiber cv. ‘Delores’ and the CBD-dominant cultivar ‘Charlotte's Web’ to be heavily galled, while the CBD-dominant cultivar ‘Wife’ had little or no galling. Nothing is known of the chemical basis in hemp for root-knot nematode resistance, and the role of psychoactive-medicinal compounds in nematode resistance is uncertain, as those compounds are found largely in the inflorescences rather than somatic tissue. However, psychoactive and other chemical compounds that have been reported in roots are active at very low concentrations and may impact the health span and life span of root-knot and other endoparasitic nematodes. More research is needed before definitive answers are available. Nevertheless, the frequent nematicidal effects of *C. sativa* plant parts (Table 1), including root extracts (Haseeb et al., 1978; Ganaie and Khan, 2016), are highly suggestive of some useful chemistry that might be exploited as biopesticides.

Another common approach in nematode management is crop rotation. Until the picture is clearer about which nematodes are likely to damage hemp, appropriate rotation schemes cannot be specified. Interestingly, hemp may have a role in rotations with soybean and other crops, as Zhang et al. (2013) demonstrated that hemp in rotation with soybean reduced *H. glycines* populations. Management of this nematode has been exhaustively studied, and a similar approach could be made in management of hemp-parasitic nematodes.

There is no evidence yet of the impact of mono-cultured hemp crops on nematode communities and their potential pest species. Therefore, multi-year field trials and samplings will be needed across a wide range of germplasm to determine what will happen in hemp fields, along with greenhouse tests of selected cultivars with the most likely pathogens, especially root-knot and lesion nematodes.

Many avenues of useful research need to be explored as hemp becomes a stable, large-acreage, reliably profitable crop. *Cannabis sativa* produces many unique, biologically active molecules that could be screened for their effects on plant pathogens (Andre et al., 2016), and more specifically on nematodes with an initial model such as *Caenorhabditis elegans*. Although roots have been used for centuries as sources for medicinal compounds, their chemistry is poorly understood, and research on chemistry of roots has lagged that of inflorescences.

Surprisingly, there appears to be nothing published about the anatomy of hemp roots, much less nematode development within them. The presence of fibers in hemp roots (R. N. Trigiano and E. C. Bernard, unpubl.) leads to a scenario that early development of fibers may inhibit the ability of root-knot nematodes to induce giant cells and initiate cortical hyperplasia. In some cases, acid fuchsin-stained nematodes remain more-or-less vermiform far from the root tip in susceptible cultivars, indicating a failure to induce giant cells. It seems possible that the presence of fibers, if they are induced soon enough, could serve as a mechanical barrier to successful penetration to the newly forming vascular stele.
